# Immunotherapies in MuSK-positive Myasthenia Gravis; an IgG4 antibody-mediated disease

**DOI:** 10.3389/fimmu.2023.1212757

**Published:** 2023-07-26

**Authors:** Aigli G. Vakrakou, Eleni Karachaliou, Elisabeth Chroni, Vasiliki Zouvelou, Dimitrios Tzanetakos, Stavroula Salakou, Marianna Papadopoulou, Socrates Tzartos, Konstantinos Voumvourakis, Constantinos Kilidireas, Sotirios Giannopoulos, Georgios Tsivgoulis, John Tzartos

**Affiliations:** ^1^ First Department of Neurology, Medical School, National and Kapodistrian University of Athens, Athens, Greece; ^2^ Second Department of Neurology, Attikon University Hospital, National and Kapodistrian University of Athens, Athens, Greece; ^3^ Department of Neurology, School of Medicine, University of Patras, Patras, Greece; ^4^ Department of Physiotherapy, University of West Attica, Athens, Greece; ^5^ Tzartos NeuroDiagnostics, Athens, Greece; ^6^ Department of Neurobiology, Hellenic Pasteur Institute, Athens, Greece; ^7^ Department of Pharmacy, University of Patras, Patras, Greece; ^8^ Department of Neurology, Henry Dunant Hospital Center, Athens, Greece; ^9^ Department of Neurology, The University of Tennessee Health Science Center, Memphis, TN, United States

**Keywords:** Myasthenia Gravis, MuSK, IgG4, anti-CD20, FcRn, CAAR-T cells

## Abstract

Muscle-specific kinase (MuSK) Myasthenia Gravis (MG) represents a prototypical antibody-mediated disease characterized by predominantly focal muscle weakness (neck, facial, and bulbar muscles) and fatigability. The pathogenic antibodies mostly belong to the immunoglobulin subclass (Ig)G4, a feature which attributes them their specific properties and pathogenic profile. On the other hand, acetylcholine receptor (AChR) MG, the most prevalent form of MG, is characterized by immunoglobulin (Ig)G1 and IgG3 antibodies to the AChR. IgG4 class autoantibodies are impotent to fix complement and only weakly bind Fc-receptors expressed on immune cells and exert their pathogenicity *via* interfering with the interaction between their targets and binding partners (e.g. between MuSK and LRP4). Cardinal differences between AChR and MuSK-MG are the thymus involvement (not prominent in MuSK-MG), the distinct HLA alleles, and core immunopathological patterns of pathology in neuromuscular junction, structure, and function. In MuSK-MG, classical treatment options are usually less effective (e.g. IVIG) with the need for prolonged and high doses of steroids difficult to be tapered to control symptoms. Exceptional clinical response to plasmapheresis and rituximab has been particularly observed in these patients. Reduction of antibody titers follows the clinical efficacy of anti-CD20 therapies, a feature implying the role of short-lived plasma cells (SLPB) in autoantibody production. Novel therapeutic monoclonal against B cells at different stages of their maturation (like plasmablasts), or against molecules involved in B cell activation, represent promising therapeutic targets. A revolution in autoantibody-mediated diseases is pharmacological interference with the neonatal Fc receptor, leading to a rapid reduction of circulating IgGs (including autoantibodies), an approach already suitable for AChR-MG and promising for MuSK-MG. New precision medicine approaches involve Chimeric autoantibody receptor T (CAAR-T) cells that are engineered to target antigen-specific B cells in MuSK-MG and represent a milestone in the development of targeted immunotherapies. This review aims to provide a detailed update on the pathomechanisms involved in MuSK-MG (cellular and humoral aberrations), fostering the understanding of the latest indications regarding the efficacy of different treatment strategies.

## 1 Introduction

Myasthenia Gravis (MG) is the most extensively studied and best-understood autoantibody-mediated autoimmune neurological disease; it affects the endplate region of the postsynaptic neuromuscular junction. MG is diagnosed based on clinical, electrophysiological, and serological findings (1). In approximately 80% of patients with generalized MG, it is characterized by increased levels of circulating immunoglobulin G1 (IgG1) and IgG3 autoantibodies against the acetylcholine receptor (AChR) (2, 3). Less frequently, patients who lack detectable serum AChR antibodies mainly demonstrate IgG4 antibodies against muscle-specific tyrosine kinase (MuSK) or IgG1 antibodies against low-density lipoprotein receptor-related protein 4 (LRP4). A small subgroup of patients (approximately 7%) with MG has no detectable circulating antibodies (4, 5).

MuSK belongs to the receptor tyrosine kinases enriched at the neuromuscular junction (NMJ) and is important for its differentiation and development (6, 7). The ectodomain of MuSK is comprised of three immunoglobulin (Ig) -like and one cysteine-rich/Frizzled domain, while the cytosolic compartment is mainly composed of a tyrosine kinase domain (8). Agrin, a heparan sulfate proteoglycan, released from motor neurons, binds to MuSK coreceptor LRP4 (located in the muscle membrane) and this interaction leads to the generation of a hetero-tetramerized complex consisting of 2 LRP4 and 2 MuSK molecules. The 2 MuSK molecules are dimerized, and the intracellular kinase domains are activated through autophosphorylation (transphosphorylation of tyrosine residues in the cytoplasmic domains like the Y553 site) (9–11). This phosphorylation leads to the recruitment of downstream kinase-7 (Dok-7), located in the cytosol of myofibers, which is phosphorylated and activated. Dimerization of Dok-7 leads to juxtaposition of the 2 MuSK molecules required for the full activation of MuSK (10, 12). Downstream of Dok-7 activation are the adaptor proteins Crk and Crk-L that recruit further proteins like Sorb1 and Sorb-2 finally leading to the activation of the scaffold protein rapsyn that is responsible for the proper AchR clustering in the muscle membrane (13, 14). Other downstream pathways of Dok-7 activation involved in AchR clustering are the activation of Rac and Rho proteins, or disheveled 1(Dvl1) and tumorous imaginal discs protein (Tid1) (15, 16). Moreover, the overactivation of Musk is regulated by various mechanisms involving the Wnt pathway leading to endocytosis, by Dok-7 ubiquitination and degradation, or a stringent process involving the juxta membrane and the autoactivation loop autoinhibition (17, 18). Constitutively active MuSK has been shown to induce ectopic NMJ-like structures independent of Agrin *in vivo (* 19). In the research area of MuSK-MG, the activation of the agrin-LRP4-MuSK pathway is of importance as the patients circulating antibodies targeting MuSK affect MuSK phosphorylation and/or Agrin-dependent AChR clustering with complex mechanisms explained below.

In MuSK-MG, unprovoked disease exacerbations and myasthenic crises frequently occur even despite high doses of immunosuppression (20, 21). Nevertheless, in recent years, the early diagnosis of these patients and the administration of rituximab has led to a noticeable reduction in treatment-refractory cases (22). Very rare cases with mild ocular symptoms and spontaneous remission have been described (23, 24). Approximately, 10–15% of MuSK- MG patients have a refractory disease or suffer from relapses on tapering immunosuppressive medication (25). It is frequently difficult to manage this subset of individuals who do not respond favorably to steroids or standard immunosuppressants. Classical treatments for MuSK-MG include nonspecific immunomodulation (corticosteroids and plasmapheresis) in the condition of myasthenic crisis or an acute exacerbation of MG symptoms, and nonsteroidal immunosuppressants that serve as steroid-sparing agents for long-term remission. There is an urgent need for novel and more efficient therapies for MG due to devastating side effects and treatment resistance. Novel and upcoming immunotherapies for MuSK-MG include molecules that target B cells, specific B cells that recognize the MuSK antigen, plasmablasts, cytokines involved in B cell maturation, and neonatal fragment-crystallizable antagonists Receptors (FcRn) involved in the synthesis and recycling of immunoglobulins. In this review, we wanted to have an overview of these innovative therapies. In light of this, we will initially emphasize on the cellular and humoral mechanisms involved in the pathophysiology of the disease, as accomplishing this will enable researchers to create new, highly targeted medications.

## 2 General clinical aspects of MuSK-MG

MuSK-MG is a rare subtype of MG that affects 5–8% of MG patients. It usually appears alongside more severe clinical signs and has an initial start with quick progression within weeks affecting predominantly bulbar muscles and causing symptoms of jaw muscle fatigue, difficulty swallowing or speaking as well as diplopia and ptosis (26). Best clinical practice guidelines suggest that in suspicion of MG, we should test AChR antibodies first and if negative, then MuSK antibodies should be evaluated (4). Clinicians should have in mind that during the course of AchR-MG, reassessment of antibody status is advisable when there is an unexpected change of pattern of clinical involvement and alterations in therapeutic response as there are emerging cases with double positivity with MuSK at the beginning (rarer) and especially years after disease onset (27–32). Double positivity has also been observed in a drug-associated MG case under d-penicillamine treatment (33).

MuSK-MG is considered to emerge during the fourth decade of life in contrast to AChR-MG which exhibits a more bimodal appearance with many patients diagnosed before the fourth decade and many others after the sixth decade. MuSK-MG more frequently affects women, whereas AChR-MG affects young women but also older men (34, 35). The pattern of muscle involvement in MuSK-MG is more directed to the facial, bulbar, and respiratory muscles (36). Many MuSK-positive patients experience a head drop owing to neck extensor weakness, whereas AChR-MG individuals suffer neck flexor weakness. Limb weakness is usually milder and less frequent than in AChR-MG (26, 37). A characteristic of MuSK-MG is the variably observed tongue, masseter, buccinators, orbicularis oculi muscle atrophy, as well as the occasionally seen shoulder and girdle muscle atrophy (38). Significantly, early initiation of immunosuppressive treatment could restore atrophy in designated muscles (39, 40). A cardinal difference between AChR-MG and MuSK-MG is the rare participation of the thymus with either thymoma or hyperplasia, in the latter that points to the different key immunological mechanisms underlying each subgroup (41). An intriguing aspect of MuSK-MG is that the emergence of MuSK Abs along with disease clinical features can appear years after the AChR-MG onset and thymectomy (30, 42, 43). In these unusual yet existing cases, a possible explanation might be the immunological changes following thymectomy in AChR-MG. HLA alleles that are associated with the two subtypes of MG are distinct. MuSK-MG has been associated with the risk alleles belonging to HLA-DRB1, DQ1, and DQ5 locus (44–47).

Most AChR and low-density lipoprotein receptor-related protein 4 (LRP4) antibodies are of the IgG1 subtype, while MuSK antibodies are typically of the IgG4 subtype. One of their main differences is that IgG1 antibodies can activate the complement, while IgG4 affects the neuromuscular junction without complement activation or induction of antigen internalization, but involved pathogenetic mechanisms include direct inhibition of MuSK protein interaction with other proteins and particularly LRP4 (48, 49).

The levels of IgG4 MuSK antibodies, particularly targeting the Ig-like-1 domain, have been found to correlate with disease severity in MuSK-MG, something that is not evident in AChR-MG (49). Notably, titers of antigen-specific IgG4, which outnumbered IgG1, were substantially associated with indices of disease severity (50). Furthermore, it is important to emphasize that, while IgG4 is predominant at the time of diagnosis, considerable concentrations of IgG1-3 antibodies can be detected, and are less thoroughly studied (51). In recombinant human antibodies isolated from the peripheral blood of MuSK-MG, all subclasses IgG1, IgG2, and IgG4 antibodies were identified, and part of them have pathogenic potential in *in-vitro* models (52).

## 3 MuSK-MG - pathogenetic mechanisms

### 3.1 Specific role of the B-cell lineage (memory B cells, plasmablast, plasma cells)

Based on their function, B-cells and plasma cells are in the foreground of the mechanisms that orchestrate the pathophysiology of MG. New insights into their importance and their mechanistic role have emerged after MG-related immunotherapies have been developed targeting autoantibody production and silencing of other B-cell functions that seem to have equal pathophysiological importance. Specifically, regulatory B cells, also known as B10 cells because they are responsible for producing IL-10 and direct CD4 T cell differentiation and growth, appear to have an essential part in the advancement of AChR-MG and MuSK-MG. An impaired B10 population or function has been correlated with enhanced disease severity. A CD20-specific antibody treatment (rituximab; CD20 is only expressed in immature B-cells or during their differentiation and is not expressed in plasma cells) that depletes B-cells in autoimmune diseases, revealed that early recovery of the B10 population post-treatment is beneficial for patients with MG and leads to disease recession, indicating that B10-targeted treatments can possess clinical importance (53). CD27+ memory B-cells are also involved in MG, as this memory B-cell branch was suppressed in patients with AChR-MG after rituximab (RTX) treatment (54). Plasmablasts are also important for the progression of MG and represent an in-between differentiation step of B-cells (mature B-cells that have not completely become mature plasma cells). These ephemeral cells are responsible for producing high levels of autoantibodies (55, 56). The clinical importance of CD20-expressing cells, such as B-cells and plasmablasts, has been linked with drastically diminished MuSK antibody levels after B-cell depletion due to RTX treatment (52, 57). It has also been revealed that most patients with MuSK-MG who received B-cell depletion therapy (BCDT) achieved complete and stable remission, followed by subtle levels of plasma autoantibodies. In contrast, other reports have shown that patients with MG can relapse after several years (24–42 months depending on the study and the protocol used) or not respond to BCDT therapy (58, 59). In line with previous insights, in 2020, Jiang et al. revealed that a proportion of B-cell clones actually dodge and survive the BCDT, with most of them being recognized as CD20-low memory B-cells and antibody-secreting cells (ASCs). The plasmablast implication in MG progression was also confirmed by the fact that the levels of plasma MuSK autoantibodies were enhanced in patients who had relapsed. Following the previous findings, several studies have attempted to shed light on the exact population that produces the detrimental MuSK-specific antibodies. Surprisingly, B-cells and plasmablasts producing anti-MuSK autoantibodies represent almost a nugatory proportion of cells that produce these pathogenic autoantibodies. One study showed that a specific clone (2E6) of plasmablasts with CD20-low expression could be responsible for the plasma anti-MuSK antibodies. Nevertheless, taking into account that plasma anti-MuSK antibodies are polyclonal, it is currently unclear if a single antibody clone is responsible for these plasma anti-MuSK antibodies (60).

According to another study, people with MuSK-MG who relapse had marginally higher levels of CD19+ CD27hi CD38hi plasmablasts. Furthermore, patients with MG who relapsed after BCDT possessed a large percentage (25%) of plasmablasts not expressing CD20 but had a high abundance of transmembrane activator calcium modulator (TACI) and B-cell maturation antigen (BCMA) receptors and low levels of the B-cell activating factor (BAFF) receptor. This outcome might be in line with the finding that high plasma BAFF levels (a cytokine that promotes B-cell survival and differentiation into ASC) were found in patients with AChR-MG and MuSK-MG without differences in BAFF receptor levels (61–63) (**Table 1**).

**Table 1:** Cells of B cell lineage and antibodies in MuSK-Myasthenia Gravis.

		References
New immigrant and Naïve B cells	Autoreactvity due to defects in central and peripheral immune tolerance mechanisms	(64, 65)
Regulatory B cells/B10	Lower percentages in MuSK-MG	(53, 66)
	Correlate with disease severity	
	Early recovery after rituximab associated with clinical improvement	
Memory B cell	MuSK-specific memory B cells identified in MuSK- MG patients	(52)
Plasmablasts	Mildly elevated especially during disease exacerbation/relapse	(52, 56)
	Rare MuSK-specific plasmablasts	(52, 56, 57)
	Rituximab-induced incomplete depletion	(57, 67)
	MuSK-specific IgG4 plasmablasts persist in relapse after a BCDT-induced remission	(60)
	Express high levels of the receptors TACI and BCMA, low levels of BAFF-R	(60)
IgGs		
Patient serum IgG	IgG4 anti-MuSK abs: functionally monovalent due to Fab exchange, inhibit MuSK phosphorylation (*in vitro*)	(51, 68–70)
	IgG4 subclass mediates pathology by inhibiting the interaction between MuSK and LRP4 (*in vivo*)	(49, 71–74)
	IgG4 binds to the collagen tail subunit (ColQ) of acetylcholinesterase and blocks the binding of ColQ to MuSK	(48, 50)
	Unable to bind to C1q to activate the complement cascade	(69)
	Unable to bind to FcγR to activate immune cells	(69)
	Passive transfer of IgG4, but not IgG1-3, into mice induces experimental gravis	(73)
	IgG1-3 induce complement activation ad MuSK endocytosis	(75, 76)
Recombinant Abs from memory B or ASC cells	Belong to various subclasses, MuSK IgG1-3s exhibit pathogenesis *in-vitro*	(52, 73, 75–77)
	MuSK-specific rAbs recognized epitopes within the first Ig- like domain of MuSK	(78)
	Bispecific MuSK-rAbs stimulated MuSK phosphorylation *in vitro*, while still inhibiting AChR clustering	(77)
	Rabs with pathogenic capacity derived from clonal MuSK-specific B cell clones reemerged after BCDT-mediated remission, predating disease relapse	(60)
	Exceptionally high affinities through the process of affinity maturation	(79)
	Monovalency increases the pathogenic effect of MuSK antibodies	(77, 80)

ASC, antibody-secreting cells; Abs, antibodies; MuSK, muscle-specific kinase; BCMA, B-Cell Maturation Antigen; TACI, Transmembrane activator calcium modulator, and cyclophilin ligand interactor; BAFF-R, B-cell activating factor receptor; BCDT, B cell depletion therapy; Rabs, recombinant antibodies.

Collectively, it seems that MuSK-MG tolerance defects (both in peripheral and central checkpoints) lead to the generation of autoreactive B cells (81). Moreover, apart from these aberrations in the immature and new emigrant B cells (assessed by tolerance assays and self-reactivity of produced antibodies), short-lived CD20-expressing, MuSK-specific plasmablasts seem to account in part for the autoantibody production (82). Apart from plasmablasts, memory antigen-specific B cells (recognized as CD20low memory B cells) also produce autoantibodies and these clones persist or even reemerge in the post-rituximab period (60).

### 3.2 Autoantibodies in MuSK-MG – pathogenetic role

#### 3.2.1 Emphasis on IgG4: its properties

The pathology of MuSK-MG is mainly mediated by IgG4-subclass antibodies that exert their pathogenetic action *via* the inhibition of interactions between MuSK and collagen Q or LRP4. This is achieved *via* binding to the first Ig-like domain of MuSK, leading to decreased levels of phosphorylated MuSK and subsequent reduction of both agrin-induced and agrin-independent AChR clustering and signaling (48–50). Specific high-affinity maturation processes should be performed for anti-MuSK IgG4 to reach its pathogenic potential. More specifically, MuSK recognition by the anti-MuSK antibodies allows their structural maturation and functional monovalency that stimulates their pathogenicity (79, 83). Two possible mechanisms that affect MuSK-mediated AChR clustering have been proposed; a. bivalent IgG cross-links and auto-phosphorylates MuSK rendering its activation, b. functionally monovalent IgG4 directly inhibits MuSK autophosphorylation (84). Additionally, *in vitro* experiments in C2C12 cells revealed that MuSK antibodies, Fab fragments, and IgG4, but not IgG1-3, promoted the frailty of the MuSK/LRP4 complex, reducing AChR clustering (51). Another likely explanation is that MuSK antibodies disrupt the LRP4-mediated retrograde cascade, justifying enhanced AChR dissemination and acetylcholine levels (85). This is also supported by *in vivo* experiments where MuSK-MG mice failed to exhibit presynaptic acetylcholine increase that is phenotypical in AChR-MG mice (86). The effect of enhanced pathogenicity of these autoantibodies can also be documented by the isolation of two novel MuSK monoclonal antibodies that are produced from plasmablasts detected in BCDT relapse and can bind to the Ig1-like domain of MuSK (79). The MuSK Ig-like 1 domain is known as the main immunogenic epitope when challenged with polyclonal IgG4 fractions and serum derived from MG patients (87). The Ig-like domain 2 of MuSK, which can be observed by different IgG4 and IgG3 antibodies also leads to AChR cluster inhibition, surprisingly coupled with augmented MuSK phosphorylation, signifying multiple compensatory pathways (52).

**Figure 1:**
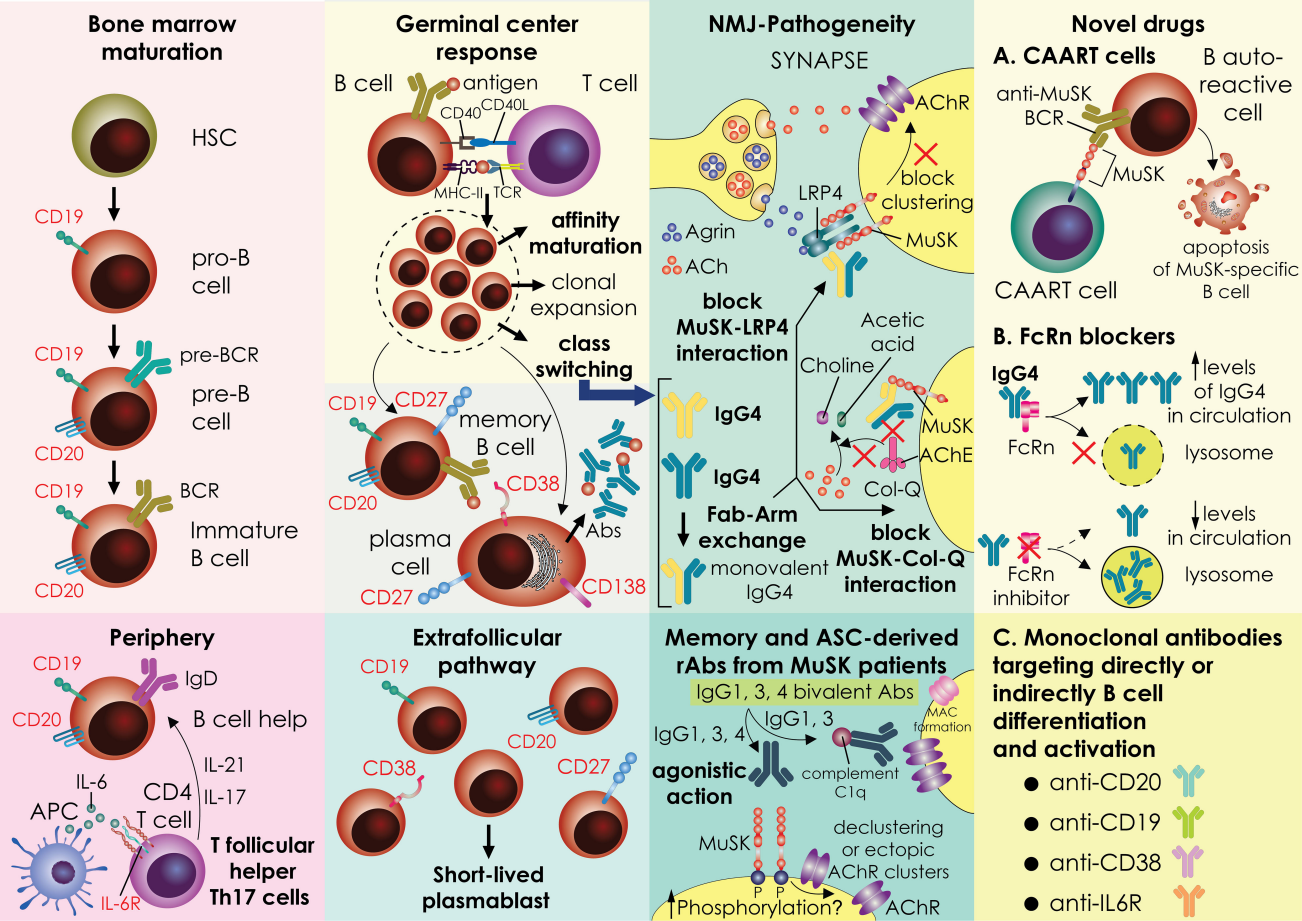
A graphical representation of a mechanistic theory for how MuSK autoantibodies are produced. The stages of B-cell development and differentiation are depicted in the image. In the bone marrow, the development process progresses through the pro-B-cell, pre-B-cell and immature-B-cell stages, and then gives rise to naïve B cells in the periphery. Naive B cells can take messages for cell proliferation and activation by CD4 T cells. specially in MuSK-MG patients, the expanded circulating T follicular CD4 T cells expressing IL-17 (Tfh17) secrete cytokines fostering B cell activation. B cells enter the germinal centers where are likely to encounter an antigen and receive support from T cells. Active germinal centers (GC) give rise to memory B cells and LLPCs, whereas the extrafollicular pathway of B cells activation ends up with the generation of SLPBs that enter the periphery (other pathways that also exist, but are not shown here, are less studied pathways). Important processes inside the germinal centers are B cell clonal expansion and affinity maturation. In MuSK-MG, a pool of memory B specific for MuSK, as well as SLPB have been recognized as projectors of cells secreting pathogenic IgG4 antibodies. In MuSK-MG, a specific cytokine milieu and other unknown factors foster IgG4 class-switching. An inherent property of IgG4 is the Fab-arm exchange that generates monovalent IgG4 antibodies that cause neuromuscular junction dysfunction by interfering with MuSK interaction with other proteins like LRP4 and ColQ. Recombinant antibodies produced by MuSK-MG patient memory or antibody-producing cells have exhibited also a pathogenicity profile with a different pattern of action (agonistic action, complement activation) from antibodies isolated from the serum of patients. A subset of recombinant antibodies produced in-vitro exhibit agonist action and enhance phosphorylation of MuSK. Nevertheless, some of them inhibit agrin-induced AchR clustering (agrin not shown). These effects need further in vivo studies for assessing antibody pathogenicity. Novel therapeutic strategies involve Chimeric autoantibody receptor (CAAR) T cells that are endogenous T cells from patients engineered in vitro to target B cells that produce autoantibodies against MuSK leading them to apoptotic cell death. MuSK-CAART is designed to specifically eliminate anti-MuSK B cell receptor (BCR) expressing B cells MuSK CAAR T cells efficiently kill various anti-MuSK BCR expressing cells but not control B cells. To achieve this, the CAAR comprises the native MuSK ectodomain tethered to tandem CD137-CD3ζ signaling domains. Another novel treatment approach currently in clinical trials for MuSK-MG is the targeting of the FcRn with inhibitors. FcRn functions as a protector of IgG from lysosomal degradation through the recycling and transcytosis of IgG within specific cells. Antagonism of this receptor promotes IgG degradation, leading to reduced overall IgG and pathogenic autoantibody levels. Finally, various monoclonals are being tested in clinical trials directly or indirectly targeting the activation and differentiation of B cells like anti-CD19, anti-CD38, and anti-IL6R. The surface expression of the proteins targeted by monoclonal is depicted in the picture. MG, MG; LLPCs; long-lived plasma cells; SLPBs, short-lived plasmablasts; ColQ, collagen-like tail subunit of Asymmetric Acetylcholinesterase; CAAR-T, Chimeric autoantibody receptor T cells; MuSK, muscle-specific kinase; BCR, B cell receptor; FcRn, neonatal Fc receptor; NMJ, neuromuscular-junction.

MuSK-specific autoantibodies dictate the pathogenic effect and can be valuable prognostic biomarkers to predict post-BCDT relapse, as they can be identified alongside MuSK-positive B-cells prior to relapse (88). Additionally, anti-MuSK autoantibodies can abrogate the formation of the complex between MuSK and the subunit (ColQ) of acetyl-cholinesterase on the presynaptic muscle membrane by binding to the latter (89). In addition, this complex formation interaction has been reported to reduce AChE at the synapse and promote ACh accumulation within the synapse (48). This biochemical cascade has been proposed to be responsible for the hypersensitivity that patients with MuSK-MG exhibit after treatment with AChE inhibitors (90). Several other reports have well described the ability of anti-MuSK antibodies to disrupt the NMJ (68, 71). This has been accompanied by the fact that functional and structural synapse homeostasis is being disrupted because of inactivated MuSK at the NMJ (72) **(** **Table 1** and **Figure 1** **).**

#### 3.2.2 Evidence of pathogenicity from animal models (immunization and passive transfer models) and recombinant Abs from patient memory B or plasma cells

The importance of anti-MuSK IgG4 antibody pathogenicity has been documented during experiments where MG was induced in mice who passively received IgG4 antibodies derived from patients with MG. The same study also implicated that the pathogenesis of MG is not mediated *via* complement-related mechanisms, as the IgG4 subclass cannot activate the complement per se (73). Two reports, where placental transfer of antibodies was able to induce transient MG in the neonate, also strengthen this hypothesis (91). Of note, interspecies mechanistic studies revealed that only the extracellular part of human MuSK protein can induce myasthenic phenotypes in both murine and rabbit NMJs, whereas murine MuSK phosphorylation is reduced when purified antibodies obtained from patients with MuSK-MG, were inoculated (49, 74, 92). More mechanistic studies on anti-MuSK IgG4 CH2-amino substitutions have revealed that these constructs failed to activate immune cells or the complement through FcγR or C1q, respectively (69). Furthermore, monoclonal MuSK antibodies derived from B-cells and plasma cells of patients with MG are able to increase MuSK phosphorylation and inhibit AChR clustering in C2C12 myotubes as expected (52, 77). Furthermore, monovalent Fab fragments produced from patient-derived bivalent monospecific MuSK antibodies can diminish MuSK phosphorylation and AChR clustering (93).

## 4 MuSK-MG as part of neurological autoimmune disorders with the characteristic presence of IgG4

MuSK-MG belongs to a wider family of neurological autoimmune disorders characterized by the presence of IgG4 autoantibodies directed against different antigenic targets, specific to each disease (IgG4-AID). It is important to discriminate these neurological autoimmune diseases from IgG4-related diseases (IgG4-RLD) that comprise multiorgan diseases hallmarked by tissue-destructive fibrotic lesions along with lymphocyte and IgG4 plasma cell infiltrates and elevated serum IgG4 concentrations (94)(Perugino CA, 2020). It is unknown if IgG4-AID and IgG4-RLD share pertinent clinical and immunopathological characteristics, but more recent data indicate that they are separate disease entities (95). Some typical examples of IgG4-AID, apart from MuSK-MG, include LGI1 (leucine-rich, glioma-inactivated-1) autoimmune encephalitis in which the autoimmune response is directed against a secreted protein that stabilizes the trans-synaptic complex (LGI1), Caspr2 (Contactin-associated protein-like 2) associated encephalitis and/or peripheral nerve hyperexcitability, nodal/paranodal chronic inflammatory demyelinating polyradiculoneuropathy with antibodies to neurofascin-155, contactin-1/caspr-1, and anti-IgLON5 (immunoglobulin-like cell adhesion molecule 5) disorder with antibodies against an adhesive protein (IgLON5) in the Central Nervous System (CNS). Specific autoantigen has not been described in IgG4-RLD. *Via* novel single-cell immunoglobulin sequencing techniques and subsequent cloning, galectin-3 was identified as an autoantigen in patients with IgG4-RLD. Anti-galectin-3 autoantibodies were found in 28% of a variety of patients with IgG4-RLD, albeit their pathogenetic function is uncertain (96).

The exact cytokine milieu permitting class switch to IgG4 in MuSK-MG is still unknown. It is known that a TH2 milieu supported by IL-4 and IL-3 cytokines along with a synergistic action of IL-10 is a significant factor for the IgG4 class switch (97–99). Nevertheless, this simplified scenario has not been verified in MuSK-MG, as IL-10 production has been found to be diminished especially by stimulated B cells. Nevertheless, other cell sources of IL-10 have not been examined especially inside the germinal centers. Previous studies have shown that IL-10, IL-6, and TNF-alpha produced by B cells are down-regulated in MG, regardless of the kind of antibodies produced (100). Therefore, B cells’ inefficient cytokine production solely cannot explain the propensity to IgG4 which is usually fueled by IL-10 and IL-6. A specific cytokine or cellular network (T helper cells, dendritic cells, etc.) in the germinal centers or in the extrafollicular compartment of MuSK-MG patients could be responsible for fostering IgG4 production. Indeed, in an animal model of MG after MuSK immunization, it was noticed the predominance of a Th2 milieu, that cannot be easily translated to human MuSK-MG (101). T-cells from MuSK-MG patients showed a higher Th1/17 versus Th2 response compared to healthy controls (102, 103). CD4+ T cells had higher IL-2, TNF-alpha, and IL-17. Patients with MuSK-MG exhibited a greater proportion of CD4+ T cells that produced combinations of IFN-gamma/IL-2/TNF-gamma, TNF-alpha/IL-2, and IFN-gamma/TNF-alpha. Interestingly, Treg numbers and CD39 expression were not different from control values. Moreover, a higher Tfh/Tfr ratio was found in MuSK-MG patients along with increased frequencies of Th17-producing Tfh cells and higher Tfh-fostered IgG synthesis (104). Indeed, MuSK patients tend to have mildly elevated levels of immunoglobulin production (IgG) compared to controls (p=0,057), something however also observed in AChR-MG (105).

One theory is that the type of antigen (time of exposure) and the specific genetic pre-disposition as mainly determined by HLA alleles, could generate the appropriate environment -genetic conditions for predisposing to a “skewed” isotype profile towards IgG4 (69, 106). Indeed, patients with DQ5+ MuSK-MG appear to have a limited oligoclonal T-cell response that is specific for MuSK, showing a shared among patients repertoire that persists even under current immunosuppressive therapies (107). Future studies will delineate if specific T-cell receptor (TCR) rearrangement under exposure to a specific antigen (MuSK or another), favors specifically IgG4 B cell production.

It is not known in MuSK-MG when during disease evolution the class switch occurs. Moreover, it is not known exactly which is the precursor subclass from which the switch begins. In-depth sequencing analysis of different B cells and antibody screening could reveal similarities (shared lineages) and common progenitors, explaining the exact route followed for the final IgG4 class switch.

On the other hand, in some of the patients with IgG4-related autoimmune neurological disorders, the IgG4 possesses pathogenic potential especially in blocking protein-protein interactions, whereas in IgG4-RLD the role of the elevated IgG4 is unclear and no specific antigenic targets have been described so far. Usually IgG4-AID serum IgG4 levels are within the normal range (a small proportion of MuSK-MG patients have elevated IgG4 levels), and the suspected HLA risk loci are unique and not shared with IgG4-RLD. Common clinical characteristics among IgG4-AID and IgG4-RLD are the moderate response to corticosteroid treatment, whereas both diseases display exceptional response to B cell depletion, especially when other immunosuppressants have failed (108). Initial therapy with glucocorticoids, and subsequent additional immunosuppressive or a biologic agent, particularly RTX, is required in most patients. Variable decrease of antibody titers after RTX therapy has been observed in autoimmune neurological disorders with antibodies against LGI1, Contactin1, NF155 (neurofascin155), DPPX (dipeptidyl-peptidase-like protein-6), and Caspr2 (contactin-associated glycoprotein2) that share the common features of the presence of IgG4 antibodies (109–114). The presence of short (SLPB)- or long-lived plasma cells (LLPB) is not widely studied, as most studies are lacking due to the rarity of these diseases in the general population. The response to RTX is variable and in most of these disorders, a considerable number of patients exhibit a favorable response (like DPPX encephalitis, LGI1, caspr2 encephalitis), whereas in some the response is less profound (like in IgLON5) (115–117). Current evidence suggests that early and short-term RTX therapy could be an effective treatment option specifically for LGI1, and caspr2 encephalitis, albeit larger studies are needed to confirm this finding (64, 118, 119). RTX therapy resulted in a substantial decrease in autoantibody titer that was associated with clinical improvement in patients with Chronic inflammatory demyelinating polyneuropathy (CIDP) with primarily IgG4 autoantibodies against NF155, contactin-1, or Caspr1 (113).

Intravenous immunoglobin (IVIG) as a therapeutic option is not widely studied in these disorders. On the other hand, more evidence exists in IgG4-AID. For example, in patients with pemphigus, high doses of IVIG have been shown to be effective, and one mechanism was postulated to be the blockage of FcR by IVIGs and increased IgG degradation including IgG4 (120). IgG4 neurofascin antibody (NF-155)-mediated CIDP is less responsive to IVIG and predominantly responsive to anti-CD20 treatment (121). Nevertheless, in MuSK-MG, as analyzed below in detail, there is a variable response in IVIG with clinical efficacy observed in 11-60% of patients (20, 21, 122–127).

IgG4 antibodies, apart from their autoreactivity displayed in specific IgG4-AID are also part of an anti-inflammatory response related to Th2-driven IgE (allergic) responses. Indeed, a considerable number of patients belonging to the IgG4 RLD have increased IgE apart from IgG4. This is not the rule in most IgG4-AID, as only mild elevations have been observed. Chronic exposure to allergens leads to upregulation of IgG4, a clinical phenomenon seen in beekeepers (128). Antigen-specific IgG4 limits IgE-mediated allergy as IgG4 competes with IgE for the same antigen having at the same time weaker effector functions such as complement mobilization, antibody-dependent cellular cytotoxicity, and inability to crosslink the antigen or engage activating Fcγ receptors on immune cells. In the case of a passive transfer mouse model of AChR-MG, IgG4 antibodies against the AChR acted in a protected mode as counterbalanced the pathogenic effects of IgG1 antibodies against the AChR (129).

## 5 Diagnostic tools for MuSK Myasthenia Gravis (electrophysiological studies and antibody testing)

The aberrant neuromuscular transmission in MG can be confirmed using two electrophysiological procedures; first, a compound muscle action potential (CMAP) amplitude/area reduction of more than 10% on low-rate repeated nerve stimulation (RNS) and second an increased jitter on single-fiber electromyography (SF-EMG) (130). The sensitivity of the RNS for MG diagnosis is less in MuSK-MG compared to AChR-MG, and this is more relevant when testing distal limb muscles (only 12%–57% of MuSK patients exhibited decrement) (21). Thus, clinicians should be aware that RNS should be applied in more proximal muscles, facial muscles, and especially to the orbicularis oculi. RNS testing of this group of muscles could increase the diagnostic sensitivity (sensitivity of 75%–85% in MuSK). Similar to RNS in MuSK-MG, SFEMG when performed on more proximal muscles, including the deltoid, frontalis, orbicularis oculi, or cervical para-spinals, is most often abnormal (reaching 100% sensitivity in facial muscles), in contrast to the regularly seen normal findings in peripheral muscles such as extensor digitorum communis (26, 130). In routine needle EMG in MuSK-MG more frequently may detect myopathic changes with scattered fibrillation potentials, whereas in AChR these features occurred after long-term treatment with corticosteroids (87, 131).

Regarding laboratory methods to detect MuSK antibodies the radioimmunoassay (RIA) which involves the immunoprecipitation of the extracellular domain of 125I-MuSK incubated with patient sera, represents the current gold standard for antibody identification in MG (132). RIA is positive for antibodies to muscle AChR in 80-85% of patients and for antibodies to MuSK in 5-7% of patients (38). To avoid radioactivity and increase sensitivity, cell-based assays (CBA) have been even more sensitive than RIA, as the antigen is expressed in a more native conformational form and more easily recognizable (133, 134). An international study examining sera from 633 SNMG (seronegative MG) patients from 13 European countries, revealed a prevalence of 13% for MuSK antibodies (5–22% depending on the country) although most of the antibodies detected were predominantly of the IgM type (135). A very recent study has shown that 8% (95% CI 2.9-16.6) of patients without MuSK (tested by RIA) or other antibodies have detectable binding to MuSK detected by CBA (performed in the human embryonic kidney, HEK293, cell line) (136). The live cell-based assay (CBA) can identify low-affinity antibodies against MuSK in a considerable subset of seronegative individuals with greater sensitivity and specificity than the RIA (133, 137). When compared to RIA, commercially available fixed CBA appears to have a stronger capacity to identify AChR and MuSK-Ab and may be beneficial as a serological test or a first diagnostic test in patients with double seronegative MG (137). Live CBA may be useful for serological evaluation of RIA and fixed CBA-negative samples (136). Although MuSK-ELISA (Enzyme-linked immunosorbent assay) is easier to be performed in a diagnostic lab, it has been shown that lacks sensitivity and specificity compared to RIA and CBA and therefore it is not highly recommended (69, 138).

## 6 Therapeutic options in MuSK Myasthenia Gravis

One of the challenges of MG is the development of effective, targeted therapies. In MuSK-MG, acetylcholinesterase inhibitors are less effective and induce frequent side effects. Therapies that target the complement are not suitable for IgG4 diseases since IgG4 antibodies do not activate the complement. That excludes eculizumab, ravulizumab, and zilucoplan from potential MuSK-MG treatments.

### 6.1 Acute treatment

Corticosteroids are the standard treatment for MG, and most patients usually respond within 2-3 weeks. MuSK-MG patients need higher doses to respond. However, about 15% of patients treated with high-dose corticosteroids respond poorly, a condition called refractory disease. This value is slightly higher than comparable data for AChR-MG (139).

Acetylcholinesterase inhibitors (AChEIs), which include pyridostigmine bromide are not well tolerated by MuSK-MG patients (causing increased side effects like fasciculations, and bronchial and oropharyngeal secretions), and in general show less effectiveness compared to AChR-MG. The intolerance of AChEIs has been shown in animal models of MuSK+experimental autoimmune MG (EAMG), in which administration of AChEIs led to denervation in the masseter muscle and neuromuscular hypersensitivity (spontaneous fibrillations) (140). Evidence from neurophysiological and histopathological findings in MuSK + EAMG mice, administration of AChEIs suggest that an excess of Ach in the synaptic cleft along with a reduction of postsynaptic AChRs due to fragmentation of AChRs and the observed down-regulation of AChE mainly affecting bulbar muscles would cause nicotinic adverse effects. Pyridostigmine clinical usage could be narrowed to a small group of patients with benign disease and without severe bulbar or respiratory symptoms (141, 142).

Plasmapheresis and IVIG are used to treat MuSK-MG with severe disease. A considerable variable response to IVIG has been observed in MuSK-MG (favorable response in 11%–61% of patients) that renders it not the preferable option in severe cases and in cases of myasthenic crisis (6, 7, 95–100). Plasmapheresis is a treatment that consists usually of 5 sessions over 1-2 weeks where the patients’ plasma is removed and replaced with healthy plasma from a donor. It is mainly used as a rescue therapy in a myasthenic crisis. IVIG infusion and plasmapheresis are considered similarly effective and fast-acting treatments for active AChR-MG. Contrary to AChR-MG, IVIG appears to be less effective than plasmapheresis in MuSK-MG; this is mostly related to the characteristics of pathogenic IgG4 antibodies and the inherent characteristics of IVIG itself (143). Of note, the VNTR2/3 genotype is suggested as a genetic risk factor for determining endogenous IgG levels and stands for poor responses to IVIG in MG patients (144). Nevertheless, this polymorphism has not yet been tested in MuSK-MG in relation to the low response to IVIG.

### 6.2 Chronic treatment

Most non-steroidal immunosuppressive therapies (NSISTs) like azathioprine, cyclosporine, methotrexate, mycophenolate mofetil, and tacrolimus have been extensively studied in AChR-MG mostly as steroid-sparing agents during prednisone tapering. Regarding their use in MuSK-MG there is less evidence and in general are more often less effective (20, 37, 90, 122, 145). Evoli et al., 2008, examined 57 MuSK-positive patients and showed that approximately 70% of patients responded well to conventional therapy with prednisone alone or along with azathioprine or cyclosporine, but 30% of them were left with permanent muscle weakness. MuSK-MG patients exhibit a lower rate of disease remission upon immunosuppressive therapy and a higher proportion of treatment dependency compared to AChR-MG (90, 122, 146). Importantly, clinicians should pay vigilance and early assess patients with MuSK-MG for poor response to initial immunotherapy, in which case they should without delay treat patients with anti-CD20, as recommended in the recently published international consensus guidelines.

### 6.3 Novel treatments

#### 6.3.1 Monoclonal and other agents affecting B cells either directly or indirectly

#### 6.3.2 Anti-CD20 agents

Rituximab is a genetically modified chimeric mouse/human IgG1-kappa monoclonal immunoglobulin. It targets CD20, a transmembrane phosphoprotein on the surface of B lymphocytes that is essential for B cell activation, differentiation, and expansion. RTX binds to CD20 and recruits immune effector cells that lead to B-cell lysis and therefore reduction of circulating CD20+ B-cells. Under normal conditions, CD20 is unique to B-cells in both humans and animals. It is first expressed by late pre-B cells in the bone marrow, mostly after Ig-heavy chain rearrangement, and its expression is reduced in plasmablasts and cells that have terminally differentiated (but not in early plasmablasts) (147).

In AChR-MG, long-lived plasma cells (not expressing CD20) are implicated in anti-AChR antibody production (148–150). Moreover, thymus-derived cells that produce pathogenic anti-AChR antibodies have been identified (151, 152); autoantibody-producing plasmablasts may possibly survive in the thymus by constitutive stimulation by autoreactive T cells. The response to RTX in AChR-MG appears to be significantly more delayed, and the autoantibody titer decline is less pronounced compared to MuSK-MG (57, 153, 154).

Most patients with MuSK-MG receiving RTX show sustained clinical improvement and a marked decline in MuSK autoantibody titer (**Table 2**). The quick and prolonged response to RTX suggests that MuSK Abs are mostly produced by short-lived Ab-secreting cells (52, 160). In contrast, bone marrow long-lived plasma cells are known to be scarcely affected by RTX (161). According to Marino et al. (2020), distinct patterns of decrease between MuSK-IgG4 and total IgG4 following RTX support the hypothesis that the majority of MuSK antibodies are produced by short-lived antibody-secreting cells (58). In particular, patients with refractory MuSK-MG that exhibited long-term remission upon RTX initiation displayed undetectable or low levels of MuSK IgG4 antibody titers, whereas total IgG and IgG4 levels transiently decreased at 2–7 months after RTX. A prior study found MuSK autoantibody-producing plasmablasts (CD27hiCD38hi B cells) upon disease recurrence in previously rituximab-induced remission patients (56). The markedly diminished MuSK autoantibody titer approximately 3 months after RTX-mediated B cell depletion suggests that RTX depletes MuSK-specific CD20^+^ memory cells and, hence, indirectly reduces short-lived autoantibody-producing CD20^-^ plasmablasts. The essential role of memory B cells in MuSK-MG is further illustrated by experiments in a mouse model of MuSK-MG, in which treatment with a low dose of teriflunomide showed to ameliorate muscle weakness, largely attributed to the suppression of memory B cells in the lymph nodes leaving unaffected the effector T cell populations (162). However, it could not be excluded the possibility that a fraction of plasmablasts could be CD20^+^ (early plasmablast, CD20^+^CD27^high^) and thus directly depleted by RTX. This model, supported by consistent findings in other IgG4-mediated diseases (163, 164), proposes that the therapeutic effect of RTX would be mostly related to the depletion of plasmablast precursors (55, 56). The effect of RTX on T-cell responses may also be relevant, with an increase in regulatory T cells observed after RTX administration in refractory MuSK-positive patients but not in AChR-positive patients (129).

**Table 2:** Studies demonstrating the effectiveness of rituximab in MuSK and AChR MG.

Study	Type of study	N of patients (AChR, MuSK, seronegative)	Type of disease	Protocol	Follow-up duration	MGFA-PIS at the end of follow-up	AChR compared to MuSK	Abs and B/T cells after treatment	Adverse events
Stathopoulos et al., 2017 (56)	Prospective study	12 (8 AChR, 4 MuSK)	MGFA class I–IIIb	375 mg/m^2^ weeks 1, 2, 3, and 4, repeat in 6 months (cycle). Repeat cycle in the case of relapse.	25–96 months	CSR, MM (3 relapsed)	In AChR MG the RTX response is more delayed and the ab titer decline is less pronounced than MuSK MG	Marked decrease of MuSK Abs titers post RTX	N/a
Zebardast et al., 2010 (155)	Retrospective study	6 (2 AChR, 4 MuSK)	Refractory MG	375 mg/m^2^ weeks 1, 2, 3, 4, 5, and 6 (1 cycle); repeat after 1 year (weeks 1, 2, 3, 4, and 5)	13-27 months	CSR, PR, MM (0 relapsed)	Both groups reduced the need for multiple- and/or high-dose immunotherapy with dramatic clinical improvement	AChR patients had a decrease in Ab titers	None
Diaz-Manera et al., 2012 (57)	Prospective study	17 patients (6 MuSK and 11 AChR)	resistant to previous therapies and MGFA class III to V	375 mg/m^2^ weeks 1, 2, 3, and 4, repeat every 2 months for 2 months, retreat if symptomatic	mean follow-up 31 months (4 – 60 months)	CSR, PR, MM (0 relapsed)	10 of the AChR patients improved but 6 of them needed reinfusions. In contrast, all MuSK patients achieved remission (4/6) or MM (2/6) status and no reinfusions were needed	Day 15 of first anti-CD20 infusion: B completely deleted, T cells unchanged Month 3: 20% decrease in IgM, no change in IgG MuSK-MG Ab titers decreased during follow-up, as early as 3 months after administration of the first dose	facial flushing and a generalized skin rush during the infusion in 2 patients
Guptill et al., 2011 (20)	Prospective multicenter review	110 MuSK (6 received RTX)	mostly MGFA III or higher	375 mg/m^2^ weeks 1, 2, 3, and 4, then repeat every 1 month for 1–2 months. Repeat in the case of relapse	average 11 years for the Rome patients, and 5.3 years for the Duke patients (range 0.5–33 years)	MM, PR, I (All 6 patients who received RTX were able to taper or discontinue all oral IS medications)	Long-term outcomes in MuSK patients are generally favorable and comparable to those of patients with AChR MG	N/a	None
Nowak et al., 2011 (156)	Retrospective study	14 (6 AChR; 8 MuSK)	Refractory generalized MG	375 mg/m^2^ weekly for 4 weeks, repeat after 6 months (2 or more cycles)	Not reported	MM or asymptomatic	Both groups respond similarly to RTX with a reduction of immunotherapy and clinical improvement	AChR Abs titers decreased a mean of 52.1% (p=0.0046) post-cycle 2.	pruritus, flushing, dyspnea, chills/rigors, leukopenia
Keung et al., 2013 (67)	Retrospective study	9 MuSK refractory	refractory MuSK-MG	375 mg/m2 weeks 1, 2, 3, and 4 (cycle), repeat every 6 months, stop at 2–5 cycles	20–66 months with a mean of 41 months	CSR, MM (0 relapsed)	N/a	Post-treatment MuSK ab status was reported negative in two patients, borderline in two patients, positive in four patients, and not available in one patient	flushing, pruritus, chills, rigors
Choi et al., 2019 (157)	retrospective study	17 (9 AChR, 6 MuSK, 2 seronegative)	Refractory MG	375 mg/m^2^ twice with a 2-week interval, followed by retreatment (375 mg/m^2^ once)	median 24 months (range 7–49 months)	11 (65%) achieved the primary endpoint, defined as the MM or better status	RTX treatment tended to be more effective in patients with MuSK MG compared with AChR MG	substantial B-cell depletion in the peripheral blood from all patients tested	infusion reactions, chest discomfort, skin rash, herpes zoster, one patient died due to thymoma
Anderson et al., 2016 (158)	Prospective, open-label study	14 (5 AChR, 6 MuSK, 3 seronegative)	Refractory MG	375 mg/m^2^ weeks 1,2,3,4 then once a month for 2 months or at a dose of 750 mg/m^2^ every 2 weeks for 1 month	(22.6 ± 2.4 months)	MMT score was greatly reduced from a baseline of 13.1 ± 1.9 (range 5–27) to 3.5 ± 0.8 (range 0–5) at the end of the study (P < 0.001)	All patients markedly improved	T-cell counts were unchanged throughout the study. CD19/CD20 cell counts were typically depleted after the first infusion of RTX (range = 6–17 days)	Post-infusion headaches
Hehir et al., 2017 (159)	Prospective multicenter review	55 MuSK (24 RTX treated, 31 control)	MuSK MGFA 3,4 or 5 in 83,4% RTX-treated and 93,6% control group	375 mg/m^2^ weekly x 4 doses. 13 were re-treated with the standard 375 mg/m^2^ weekly x 4 doses. 2 patients were treated with 1,000 mg weekly x 2	RTX-treated: 45 months (6–64, 68, 69, 71–74, 77, 79, 81–124) Control: 54 months (6–64, 68, 69, 71–74, 77, 79, 81–154, 159–196)	MGFA-PIS MM or better in final visit: RTX 67% (16/24 patients), control 26% (8/31 patients)	N/a	4 weeks after RTX cycle: B cells reached nadir 24 and 31 weeks post-RTX infusion: begin to rise	one non-responder had a side effect
Topakian et al., 2019 (166)	Retrospective study	56 (39 AChR, 14 MuSK, 3 seronegative)	Generalized MG, 14% thymoma, Severe disease defined by an MGFA-Class of IIIb or higher was present in 33 (58.9%) patients, Occular (13%)	Majority 2 × 375 mg/m^2^ within 1–2 weeks	10-53 months (median 20)	10 Remission	Remission was more frequent in patients with MuSK vs. AchR (71.4% vs. 35.9%, p = 0.022)	65% slight decline of Abs levels (unrelated to outcome)	infusion reactions, respiratory tract infections, chronic pain syndromes, enteritis, herpes zoster, erysipelas, cholecystitis, unspecified mental disorder, alopecia areata
Dos Santos et al., 2020 (197)	Retrospective multicenter study	29 (20 AchR, 5 MuSK, 4 seronegative)	MGFA > II: Refractory or steroid dependent, generalized	Various protocols	Mean 20.06 (0.17–68.93) months	In all: R 42% MM 25% Total 86%	MuSK vs AChR 75% vs. 93% had ‘improved’ response or better (CSR, PR, or MM)	N/a	infections (21.4% of patients); infusion reaction (7%); bradycardia (3.7%); and cytopenia (7%)
Meng et al., 2022 (172)	Retrospective observational study	8 MuSK	MGFA II, III, or IV	Low sose protocol: 375mg/m^2^×2 with 2 week-interval or 375 mg/m^2^ as a single infusion (1-4 cycles)	8-29 months (median 25,5)	CSR had been achieved in one patient, PR in three patients, MM in three patients and I in one patient based on the MGFA-PIS criteria	1 Improvement, 3 PR, 3 MMS, 1 CSR (Steroid dose reduction 50-100%)	at 1 month: CD19+ B-cell depletion, CD19+CD27+ B cells < 0.05% at 3 months: CD19+ B-cell < 1%, CD19+CD27+ B cells; 0% at 6 months: CD19+ decreased to 0.300%, CD19+CD27+ B cells reappeared in 3 patients	1 patient reported minor post-infusion malaise.
Heckmann et al., 2022 (198)	Prospective study	17 (5 MuSK, 10 AChR, 2 seronegative	Refractory MG, MGFA II, III, IV, V. 65% MGFA class III or IV. 3 patients MGFA class I	Single infusion: 375 mg/m^2^	Median 18 months (IQR 12; 27)	Thirteen individuals responded to a single RTX infusion including all five MuSK, 7 of 10 AChR, and one of two seronegative	Three (60%) MuSK-MG and three (30%) AchR-MG achieved asymptomatic status.	4,2 months after infusion: successful depletion of CD19+ cells in 15 from 17 patients (including 4 clinical non-responders)	No adverse effects
Zhou et al., 2021 (173)	prospective, open-label, self-controlled pilot study	12 MuSK	MGFA Class IIIb-Ivb	Single infusion: 600mg over 2 consecutive days, 100 mg on day 1 and 500 mg on day 2	6 months	6/12 asymptomatic	N/a	circulating CD19+ B cells, CD19+CD27− naïve, and CD19+CD27+ memory B cells declined by 92.0%, 90.8% and 93.9%, respectively CD3+ T, CD4+ T, CD8+ T, and natural killer cells; no difference before and after RTX CD19+ B cells, CD19+ CD27− naïve B cells, and CD19+ CD27+ B cells correlated positively with clinical scale scores The titers of MuSK abs mildly decreased after 6 mo. IgG1, IgG2, IgG3, and IgG4 levels were not significantly altered. Slight increase in three clinically improved patients.	No serious side effects
Marino et al., 2020 (58)	Retrospective study	9 MuSK refractory	Refractory, MGFA IIIb or V	375 mg/m^2^ once a week for four consecutive weeks, plus a single dose of 375 mg/m^2^ after 3 months.	17 months - 13 years	1 patient did not respond. Optimal response(MM, CSR, PR) to RTX was recorded in 6 of 9 patients (66.6%). Prednisone was tapered off in 2 patients and was reduced by 75% to 87.5% of the pretreatment dosage in the others	N/a	at 2–7 and at 12–30 months post-RTX: marked reduction of MuSK Abs at 2–7 months post-RTX: total IgG and IgG4 transiently decreased Non-responsive patients, MuSK-IgG and MuSK-IgG4 remained unchanged	No infusion reactions or long-term side effects
Litchman et al., 2020 (165)	Single-center retrospective study	33 (17 AChR, 16 MuSK)	Generalized MG median baseline MGFA Clinical Class was II	4 weekly infusions of 375 mg/m^2^ (one cycle). The interval between cycles was 6 months. Majority received 2-4 cycles	mean follow-up of 1861 ± 953.4 days.	21 patients achieved clinical remission (12/17 AChR, 9/16 MuSK) improved from a median baseline class of II to asymptomatic	MM or better at last visit: AChR 11(65%), MuSK 12 (75%)	N/a	none reported
Caballero-Ávila et al., 2022 (199)	Retrospective, observational study	30 patients (18 AChR, 12 MuSK)	Refractory MG	375 mg/m^2^ weekly for 4 weeks and then monthly for 2 months. Additional infusions in patients who relapsed	mean 85.5 (SD=48) months	18 patients achieved MM or remission	All 12 MuSK patients but only 6 (33%) AChR patients achieved MM or remission	24–48 months of follow-up: 37% of patients; hypogammaglobulinemia, 70%; mild and 30%; moderate. Median pre-treatment IgG levels: 10.7 and 9.1 g/L at 3–15 months after treatment initiation and 8.5 g/L at 24–48 months post-treatment.	Severe infections
Castiglione et al., 2022 (200)	Retrospective study	16 patients with MG (8 AChR and 8 MuSK)	refractory generalized MG MGFA clinical class III or higher	(1) 3 weekly cycles [dose = 375 mg/m^2^, (2) 2 cycles (dose = 375 mg/m^2^) separated by a 2-week interval, and (3) 2 cycles (500 mg/m^2^) separated by 2 weeks.	mean 39.1 ± 25.1 months	All 16 went into clinical remission, MGSTI level 2 or better (MM or PR either with low-dose dual therapy or high-dose monotherapy	Goal was reached significantly faster in the MuSK group although baseline MGFA was higher	6 and 12 months after induction: CD19+ and CD20+ B-cell counts remained undetectable 24 months after induction: 5 patients; undetectable counts, 6 patients; normal levels	No serious adverse effects were reported
Afanasiev et al., 2017 (201)	Retrospective, monocentric study	28 (21 AChR, 3 MuSK, 4 seronegatives)	Refractory MG	1000 mg on day 1 (D1) and D15, or 375 mg/m^2^ on D1, D7, D15 and D21. The regime is followed by a maintenance treatment, 1000 mg or 375 mg/m^2^ infusion, every 6 months	mean follow-up 27.2 months (range: 6–60 months).	At M12 (month 12), only 2 patients remained in class IV and 12 were in class II. At M36, among 12 patients, only one was in class IV	Among the 21 AChR+ patients, 12 were I, 8 were U and 1 patient was W according to the PIS. At M18, 2 AChR patients presented an exacerbation. All 3 MuSK patients did not respond to RTX (U PIS status). 3 seronegative were considered improved and 1 did not respond to RTX	N/a	11 patients (39%) had benign side effects: bronchitis, flu-like syndrome, immediate hot flashes, and paresthesia. 4 patients (14%) presented severe side effects: 1 transient aseptic neutropenia, 1 episode of paroxystic atrial fibrillation,1 infectious pneumonia, 1 histologically confirmed cerebral PML 5 years after the end of the study follow-up
Cortes-Vicente et al., 2018 (59)	retrospective, observational, multicenter study	25 MuSK	Patients with MGFA IV or V and no response to prednisone	1: 375 mg/m^2^ for 4 weeks, then monthly for 2 months, 2: two 1g doses 2 weeks apart, 3: 375 mg/m^2^ for 4 weeks	5.0 years (SD 3.3)	All patients achieved MM or a better MGFA PIS and a long-lasting response after an extended follow-up	N/a	N/a	No patient presented severe adverse events
Beecher et al., 2018 (202)	Prospective, open-label study	22 patients (10 AChR, 9 MuSK, 3 seronegative)	refractory MG	Various dosages as induction and maintenance doses if relapse occurred	mean follow-up of 28.8 ± 19.0 months (range, 6–66)	MMT scores demonstrated a reduction from a mean of 10.3 ± 5.6 to 3.3 ± 3.1 (P < 0.0001).	AChR patients showed mean MMT reduction from 10.3 ± 5.1 to 5.5 ± 2.6 (P = 0.018), whereas MuSK patients demonstrated mean MMT reduction from 10.0 ± 3.6 to 1.1 ± 2.0 (P < 0.0001).	4 of 10 relapses were predicted by CD19/CD20 recovery 3 patients showed prolonged CD19+ depletion (range, 24–45 months) after just 1 cycle	No serious adverse events occurred
Chan et al.f, 2019 (203)	Retrospective study	38 patients (AChR: 28, MuSK: 6, seronegative 4)	mostly refractory MG with MGFA class II, III, IV, V	cycles of 1000 mg, 500 mg, or 2000 mg	mean 55 months (range 1–131 months, median 49 months)	28 patients experienced clinical improvement (10 patients CSR, 7 patients MM, and 11 patients I). 5 patients were U or W. 5 patients were deceased.	N/a	N/a	5 patients died due to malignancy, infection, and asystole

Abs, antibodies; AChR+, acetyl choline receptor antibody positive; CSR, complete stable remission; I, improved; IQR, interquartile range; MGFA, MG Foundation of America clinical classification; MGSTI, Myasthenia gravis status and treatment intensity; MuSK, muscle specific kinase antibody positive; MM, minimal manifestations; MMT, Manual muscle testing; N/a, non-applicable; PIS, postintervention status classification; PR, pharmacologic remission; RTX, rituximab; SD, standard deviation; U, unchanged; W, worse; PML, Progressive multifocal leukoencephalopathy; SD, standard deviation; D, days; M, months.

Litchmann et al. (2020) conducted a single-center, retrospective cohort study comparing responses to RTX between patients with AchR-MG (n = 17) and MuSK-MG (n = 16) (165). The response was evaluated using the Myasthenia Gravis Foundation of America (MGFA) classification which aims to divide patients into categories based on disease severity and symptom localization. This study found significant clinical responses in both study groups, with MuSK-MG showing a greater benefit. The proportion of patients who achieved MGFA post-intervention status of minimal manifestations or better 12 months after initiating RTX was 58.8% in AChR-MG (n = 17), compared to 68.8% in MuSK-MG. Both groups managed to lower the mean prednisone dose at 12 months at the final follow-up compared to baseline (i.e., before RTX), with MuSK-MG displaying the lowest mean levels of prednisolone. The mean number of disease exacerbations in AchR-MG (n = 17) was 1.7 (SD = 1.2) and in MuSK-MG (n = 16),1.4 (SD = 1.1).

Topakian et al. (2019) (166) evaluated the efficacy and safety of RTX in patients with refractory MG (N = 56, 39 patients were anti- AChR antibody positive, 14 patients were anti-MuSK antibody positive, and 3 patients were seronegative). The authors discovered that patients with MuSK antibodies had a higher rate of remission than those with AChR antibodies (71.4% vs. 35.9%, p = 0.022) and reported no major safety concerns about RTX. Another multicenter, blinded prospective review research investigated the effectiveness of RTX in 55 individuals with MuSK-MG, 24 of whom were treated with RTX and 31 of whom were not. Primary objectives were attained by 58% of MuSK-MG patients treated with RTX compared to 16% of the control group (those untreated with RTX). Furthermore, at the completion of the study, 29% of the RTX-treated patients were using steroids at an average dose of 4.5 mg/day, but 74% of the controls required larger doses (prednisone mean dose of 13 mg per day) and used more immunosuppressants (159).

Marino et al. (2020) (58) performed a single-center, cohort study that investigated the effect of rituximab treatment in patients (N = 9) with refractory MuSK-MG. This study found that RTX is safe and has a long-term benefit associated with a strong steroid- and immunosuppressant-sparing effect, with 66.9% of patients achieving an optimal response with MGFA post-intervention status of minimal manifestations or better along with a 50% reduction of steroid dose, withdrawal of immunosuppressants, and no need for plasma exchange or IV immunoglobulins. A significant reduction in MuSK-IgG4 antibody levels in RTX-treated patients that were in clinical remission with sustained improvement was observed (58). This is not observed with RTX treatment in AChR-MG (167, 168).

Evidence from meta-analyses favors the use of RTX in all MG subtypes, particularly in MuSK-MG. A recent metanalysis including a total of 24 studies involving 417 patients (242 were AChR-IgG positive, 155 were MuSK-IgG positive, and 20 were defined as “double seronegative”) showed that 64% (95% confidence interval, 49-77%) of patients under treatment with RTX achieved minimal manifestation status or improved quantitative MG (QMG) score. QMG score measures the severity of MG based on clinical findings and symptoms. The estimated reduction of the QMG score was 1.55 (95% confidence interval, 0.88-2.22). Approximately 81% of patients discontinued oral immunosuppressants and this was relatively independent of the dosing. Subgroup analyses showed that the group of patients with the greater benefit belonged to the MuSK-MG group. Pooled analysis showed that 10.7% of patients experienced infusion reactions, 5.8% developed infections, and 1.9% developed hematological disorders. Only one patient (0.2%) was histologically diagnosed with progressive multifocal leukoencephalopathy (PML) (169). Another systematic review evaluated the effectiveness and safety of RTX in 99 patients who had AChR positivity and 57 patients who had MuSK positivity. The results revealed that MuSK-positive patients responded better (72% attained mild manifestation status or remission) than AChR-positive patients (30%) (168).

The most generally used rituximab administration regimens include 2 doses of 1 g 2 weeks apart or 375 mg/m^2^ infusions repeated weekly for 4 weeks. According to published data to date, there is no guideline for the proper timing for reinfusion and this is mainly based on clinical symptoms and less frequently on B cell repopulation kinetics. One study comparing various treatment protocols of RTX administration showed that patients treated with a sole induction regimen of RTX following the protocol 4 + 2 (375 mg/m^2^ every week for 4 consecutive weeks and then monthly for the next 2 months) displayed a minimal rate of clinical relapse and long‐lasting response. Authors suggested re‐treating patients with RTX in cases of clinical relapse or remaining symptoms e.g. after 4 to 6 months in order to avoid adverse events (59). Other studies tried to identify risk factors for relapse and found that CD27 memory B cells rise during relapse and this knowledge could be used for determining anti-CD20 re-administration (170). Therefore, in patients at high risk of relapses, the RTX frequency can be narrowed by monitoring the re-emerging CD27+ memory B cells (mainly CD19+CD27+), in combination with clinical assessment. The cut-off value of 0.05% CD27+ memory B cells in peripheral blood was arbitrarily used according to the experience from another autoantibody-mediated disease especially neuromyelitis optica (NMO) (171). Re-emergence of CD27+ cells was noticed with a mean time of detection for memory B cells to be 7.5 months (3 − 12). We should have in mind that a small proportion of patients may have depleted memory B cells for longer time than 12 months. The need for multiple re-doses of rituximab in MuSK-MG was assessed by Hehir, et al., in a multicenter, blinded, prospective study that include 54 MuSK patients with a median follow-up duration of 3.5 years. Importantly, 73% of patients who received multiple courses of RTX achieved the primary outcome vs 33% who received a single course. So, this study was the first to demonstrate the need for repeated doses for long-term remission (159). This is important as this implies that there is a heterogeneity in response to RTX implying district B cell pathologies in various patients.

Low-dose alternative regimens of 375 mg/m^2^ given 2 weeks apart or 600 mg single-dose inductions have been tested with satisfactory results, but these studies did not include many patients, and no control arm with a high dose was included. Nevertheless, these approaches could be applied to high-risk patients for adverse effects from prolonged B cell depletion. In the study of Meng X., et al., 2022 8 MuSK-MG patients were included, and low-dose RTX (375 mg/m^2^ for 1 or 2 infusions) was administrated and after a follow-up of 8 to 29 months found to be effective and safe. The authors noticed that three patients with CD19+CD27+ B-cell counts of >0.05%, but CD19+ B-cell counts <1% at 6 months, did not relapse. One patient relapsed among three patients whose CD27+ B-cell counts were >0.05% at 6 months. On the other hand, clinical relapses occurred even though B cells were depleted (172). Collectively, we could not reach a definite conclusion regarding the B lymphocytes number in patients and the recurrence of clinical symptoms. Another study assessing low-dose rituximab was performed by Zhou et al., 2021 (600 mg over 2 consecutive days, 100 mg on day 1 and 500 mg on day 2) (173). The titers of MuSK antibody decreased after 6 months, yet the levels of immunoglobulins, including IgG1, IgG2, IgG3, and IgG4, were not significantly altered by RTX. Interestingly, the MuSK antibody titer showed even a slight increase in three clinically improved patients. This was not in line with the previous reports possibly reflecting differences in dosage but also pointing out that in some patients other antibody-independent mechanisms of B cells could account for clinical remission (57, 58).

Rituximab should be used as soon as possible to treat MuSK+ individuals who do not react well to first immunosuppressive therapy, according to current consensus advice. In refractory AChR-MG patients, it is recommended only if other immunosuppressants resulted to be ineffective or scarcely tolerated since its efficacy is uncertain. In contrast to the common view formed by clinical practice, a phase 2 Trial of RTX in AChR - Generalized MG (The BeatMG Study), showed that RTX (every 6 months for 2 cycles) as an add-on treatment in mild to moderate AChR-MG failed to confer a significant steroid-sparing effect on chronic immunotherapy at 1 year. Additionally, no noticeable shifts in the severity of disease outcomes were found (174). However, the authors of the BeatMG study admitted that the relapse rate was reduced in the RTX group and that no conclusion can be drawn for more severe disease or MuSK-MG. According to retrospective cohort research that examined the role of RTX in patients with recently diagnosed generalized MG, RTX (usually 500 mg every six months) was associated with longer-lasting remissions than conventional immunotherapies. The RINOMAX randomized clinical trial evaluating the efficacy and safety of RTX as an adjunct to standard of care for MG patients with short onset (less than 12 months) and a Quantitative MG (QMG) score of 6 or greater recently announced that the proportion of patients with minimal disease manifestation who required only low doses of corticosteroids (a single 500 mg intravenous infusion) and did not require rescue therapy at 4 months was 71% on RTX versus 29% on placebo, showing a significant difference (175). So, the future guidelines for AChR-MG may be adapted according to new studies that have emerged. An unanswered question remains its role as an initial agent in AChR-MG, and the definition of preferred induction and maintenance doses. All studies with a considerable number of patients assessing the role of RTX in MuSK-MG, as well as the differential response among MuSK-MG and AChR-MG, are depicted in **Table 2**.

#### 6.3.3 Anti-CD19

Monoclonal antibodies targeting CD19 (like inebilizumab, a humanized IgG1κis, glycoengineered, afucosylated antibody) that are expressed in pre-B and mature B cells are promising in autoantibody-mediated diseases. CD19 is also expressed in late-stage memory B cells and circulating plasma cells. An ongoing trial, NCT04524273, tested its efficacy in moderate to severe AchR-MG or MuSK-MG (**Table 3**). Inebilizumab (MEDI-551) has been approved in neuromyelitis optica spectrum disorder (NMOSD) an antibody-mediated demyelinating disease following a phase II/III placebo-controlled clinical trial (NCT02200770). Of note, anti-CD20 treatment exhibits good clinical efficacy in NMOSD, although the therapeutic benefit does not correlate with a decrease in NMO-IgG levels in the blood of all patients that are mainly of the IgG1 subtype (176). It is important to point out that in NMOSD SLPB has been reported to be augmented during relapses, as shown in MuSK-MG, but LLPB—not found so far in MuSK-MG—is thought to participate in NMOSD pathophysiology (177–179).

**Table 3:** Novel treatment in MuSK-MG ongoing or with preliminary results.

Agents (general categories)	Targeted pathway	Product type	Study name	Reference (were available)	Number of participants with MG in general (MuSK positive were available)	Results	Study type	Study stauts	Adverse events
Abs blocking B cell lineages									
Inebilizumab anti-CD19 (MEDI-551)	pro-B cells, pre-B cells, and some plasmablasts and plasma cells.	a humanized, afucosylated IgG1κ monoclonal antibody	NCT04524273	N/A	82 MuSK-Ab+	pending	Phase III trial	ongoing	pending
Abs blocking plasma cells									
Mezagitamab anti-CD38 (Tak-079)	plasma cells, plasmablasts, and natural killer cells, some activated T and B cells, CD38 is an integral membrane glycoprotein, present in early B and T cell lineages and activated B and T cells but not in mature resting peripheral lymphocytes	humanized, IgG1	NCT04159805	N/A	36 total	pending	Phase II	Completed (no post of results)	pending
Tolebrutinib (SAR442168)	BTK inhibitor (Bruton’s tyrosine kinase)	irreversible selective inhibitor of Bruton’s tyrosine kinase (BTK)	NCT05132569	N/A		pending	Phase III	Recruiting	pending
Bortezomid	26S proteasome complex inhibitor	reversible inhibitor of the 26S proteasome complex in mammalian cells	N/A	Schneider-Gold C, et al., 2017 (180)	1 MuSK-Ab+	in combination with RTX, an effect strong enough to achieve long-term stabilization of MG	Case report	N/A	sensorimotor polyneuropathy
Indericter B cell targeting									
Satralizumab anti-IL-6	Block IL-6 promoting B cells activation and proliferation	humanized monoclonal antibody	NCT04963270	N/A	240 total	pending	Phase III	Recruiting	pending
Tocilizumab	Block IL-6 promoting B cells activation and proliferation	IgG1 humanized monoclonal antibody	NCT05067348	N/A	64 total	pending	Phase II	Recruiting	pending
Belimumab anti-BAFF	Block BAFF promoting B cells activation and proliferation	fully-humanized monoclonal antibody	NCT01480596	Karen Hewett, et al., 2018 (204)	40 total (2 MuSK-Ab+, placebo group)	completed	Phase II	completed	infections, gastrointestinal side effects nausea, influenza, and post-infusion systemic reactions
Telitacicept (RC18)	Inhibition of BAFF and APRIL	fully human TACI-Fc fusion protein	NCT04302103	N/A	29 total	pending	Phase II	Active, not recruiting	pending
T cell activation targeting									
Iscalimab anti-CD40	Fc-silenced, IgG1 mAb that blocks the CD40 signaling pathway by binding with its ligand (CD154)	IGg1, fully-human, pathway-blocking, non-depleting ab	NCT02565576	N/A	44 total	unpublished results indicate that the outcome measure of significant improvement in MG scores was not reached, although there were no safety concerns	Phase II	completed	pending no safety concerns till now
FcRn blocking abs									
Efgartigimod	FcRn inhibitor	antibody fragment that binds to the neonatal Fc receptor (FcRn)	NCT03669588), NCT04735432	Howard et al., 2021 (184)	6 MuSK-Ab+ (3 on placebo)	All six patients were MG ADL responders in cycle 1. Approved in the US and Europe for the treatment of anti-AChR+ gMG (Vyvgart^®^).	Phase II, Phase III	completed	upper respiratory tract infections (>1/10) and urinary infections, bronchitis, myalgia, and procedural headache (≥1/100 to <1/10)
Efgartigimod PH20 SC	FcRn inhibitor	antibody fragment that binds to the neonatal Fc receptor (FcRn)	NCT04735432	N/A	111 total	pending	Phase III	Completed (pending results)	pending
Rozanolixizumab	FcRn blocker	a humanized high-affinity anti-human neonatal Fc receptor (FcRn) monoclonal antibody (IgG4)	NCT03052751, NCT03971422, NCT04124965, NCT04650854	Bril et al., 2021 (185), Bril et al,. 2023 (187)	From published studies: 43 (1 MuSK-Ab+), 200 (21 MuSK+), respectively.	Phase III: all five (100%) MuSK autoantibody-positive patients in the rozanolixizumab 7 mg/kg group and all seven (100%) patients in the rozanolixizumab 10 mg/kg group were MG-ADL responders, compared to one (14%) of seven in the placebo group	Phase II, III	completed phase III and ongoing phase III	headache, back pain, diarrhea, pyrexia
Nipocalimab (or M281)	FcRn blocker	aglycosylated immunoglobulin (Ig)G1 monoclonal antibody (mAb)	NCT04951622	N/A	190 total	pending	Phase III	Recruiting	pending
Batoclimab	FcRn blocker	fully human anti-FcRn mAb blocking FcRn-IgG interactions	NCT05403541	N/A	210 total	pending	Phase III	Recruiting	pending
CAR T cells									
MuSK-CAART	targets MuSK expressing B cells	Autologous Muscle-specific Tyrosine Kinase Chimeric Autoantibody Receptor T Cells (MuSK-CAART)	NCT05451212	Oh S,et al., 2023 (205)	24 total	pending	Phase I	Ongoing	cytokine release syndrome
Descartes-08 CAR T-cells	targets B-Cell Maturation Antigen (BCMA)	Autologous T-Cells Expressing A Chimeric Antigen Receptor Directed to BCMA In patients With generalized MG	NCT04146051	N/A	30 total	pending	Phase Iib	Ongoing	pending
Haemapoietic stem cell therapy (HSCT)									
HSCT	Haemapoietic stem cell therapy	Infusing autologous stem cells to reconstitute a more tolerant immune system	N/A	Beland B, et al., 2023, (206) Burt R, et al., 2004 (207)	4 MuSK-Ab+	The average worst MG-ADL scores improved from 14.7 before to 0.3 after HSCT. The mean worst MG-QoL15 scores improved from 26.7 to 0.	Case series	completed	a tolerable side effect profile

ab, antibody; APRIL, a proliferation-inducing ligand; AChR+, acetylcholine receptor antibody positive; BAFF, B-cell activating factor, CAAR, chimeric autoantibody receptor; CAR, Chimeric antigen receptor; FcRn neonatal Fc receptor; HSCT, haematopoietic stem cell transplantation; IL-6, interleukin 6; MG, MG; MuSK+, muscle-specific kinase antibody positive; BTK, Bruton’s tyrosine kinase; BCMA, B-Cell Maturation Antigen; TACI, Transmembrane activator calcium modulator and cyclophilin ligand interactor; MG-ADL. Myasthenia gravis Activities of Daily Living; MG-QoL15, Myasthenia Gravis Quality of Life 15-item Scale; BCMA, B-Cell Maturation Antigen; RTX, rituximab (207)

#### 6.3.4 Anti-CD38

By directly targeting antibody-secreting phenotypes (plasma cells) with the anti-CD38 antibody daratumumab, novel treatment approaches aim to bypass the limitations of depletion of only CD20-expressing B cell subsets. Mezagitamab, a fully humanized anti-CD38 monoclonal (NCT04159805, ongoing), is currently being tested in MuSK-MG and the trial is ongoing.

#### 6.3.5 Proteasomal inhibitors

Proteasomal inhibitors like Bortezomib. Only one case report exists with the response of a refractory MuSK-MG patient to bortezomib (180). Nevertheless, no clinical studies are ongoing and the trial in AChR-MG was terminated due to recruitment failure.

#### 6.3.6 Others BAFFR, CD40, IL-6

Studies on belimumab (Benlysta, GSK), Iscalimab (anti-CD40) on MuSK-MG failed to show benefit in phase II trials, whereas studies on IL-6 pathway blockage are ongoing.

### 6.4 Monoclonal antibodies against FcRn receptors: rozanolixizumab, efgartigimod

FcRn inhibitors, which prevent FcRn from interacting with IgG, influence IgG breakdown and clearance and thus are used as treatments for IgG-mediated disorders (181). The principle of action of FcRn, the neonatal Fc receptor, is that it binds to the Fc region and rescues IgG from lysosomal acidic degradation, thus promoting recycling. The mechanism of action is highly reminiscent of the action of IVIG as it is known that IVIG, apart from providing anti-idiotypic antibodies and thus protecting against the action of pathogenic ones, it saturates FcRn binding and directs the autoantibodies into the degradation pathway (143, 182). Likewise, FcRn inhibitors lower IgG levels, as seen with plasmapheresis.

Efgartigimod a human IgG1 antibody Fc fragment, is the first FcRn inhibitor approved for AChR-MG (183, 184).

The initial phase 2 trial of efgartigimod, which did not include MuSK-positive patients and recruited 24 AChR-MG (1:1 randomized to IV efgartigimod or placebo), showed a rapid onset and strong clinical improvement assessed by efficacy scales [patient reported MG-ADL and physician-reported (QMG) scales]. The drug was well tolerated, with the most common adverse events being headache and mild hematological changes in the monocyte number. Importantly, clinical improvement lasted at least 6 weeks in a high proportion of patients, reminiscent of the effectiveness of Plex, which peaked at around 6 weeks. A rapid and large decrease in total IgG and IgG subtype levels was observed in all 12 efgartigimod-treated patients, peaking 1 week after the fourth infusion (approximately 70%).

A phase 3 study of the efficacy and safety of efgartigimod (ARGX-113) generalized MG included 129 (77%) AChR-positive and 38 (23%) AChR-negative patients, six of whom (4%) were MuSK antibody positive. Efgartigimod (10 mg/kg) was compared to a matched placebo and initially administered as four infusions per cycle (one infusion per week), with the option to repeat the same cycle based on the observed clinical response. All six enrolled patients were responders as assessed by the MG Activities of Daily Living (MG-ADL) scale in Cycle 1. Extension of this ongoing open-label extension study will provide more conclusive results. In general, treatment with efgartigimod was well tolerated and effective in all patients with generalized MG. Over 50% of patients responded well from Cycle 1 within 2 weeks of treatment (2-point MG-ADL improvement sustained for 4 weeks). As expected, efgartigimod resulted in a similar reduction in acetylcholine receptor autoantibodies as IgG (without effect on other immunoglobulins), and most importantly this was accompanied by concomitant improvements in symptoms. This mode of action resembles that of plasma exchange, a treatment that removes autoantibodies and is considered highly efficacious in MuSK-MG, rendering FcR blockers a highly promising treatment modality for MuSK-MG (184). Vyvgart™ received the first U.S. Food and Drug approval for AChR-MG in 2021.

Forty-three patients with gMG enrolled in a phase 2 randomized controlled trial to evaluate the efficacy and tolerability of subcutaneous (sc) rozanolixizumab (7 mg/kg), another humanized monoclonal antibody to neonatal Fc receptors. Subcutaneous administration proved to be safer compared to iv. This study included only one MuSK-positive patient, so it is underpowered due to the low number of patients. The primary endpoint (change in Quantitative MG (QMG) score from baseline to Day 29) showed no significant improvement, but when all predefined efficacy parameters (QMG, MG-ADL, and MGC) are taken into account, the data suggest that rozanolixizumab may offer clinical benefit to patients with moderate to severe gMG. There was a higher frequency of headaches (57.1%) compared to placebo (13.6%) (185). Again, this treatment led to a reduction in IgG concentration in one week that returned to baseline by 2 months, as shown in the phase I study of the drug (186).

The Phase III clinical study NCT0397142, a randomized, double-blind, placebo-controlled study evaluating the efficacy and safety of rozanolixizumab in adult patients with generalized MG, has been completed and enrolled patients with confirmed positive records of AChR or MuSK at screening (200 total MG participants). In the primary endpoint, rozanolixizumab significantly reduced MG-ADL from baseline to Day 43 (185). Very recently the results from a randomized, double-blind, placebo-controlled, adaptive phase 3 study, named MycarinG were published. 200 patients were enrolled, among which 21 MuSK-positive were included. Patients were stratified into the placebo group (12% MuSK+), 8% received rozanolizizumab 7 mg/kg and 12% received rozanolixizumab 10 mg/kg (subcutaneous infusions). With the available data, all five (100%) MuSK autoantibody-positive patients in the rozanolizizumab 7 mg/kg group and all seven (100%) patients in the rozanolizizumab 10 mg/kg group were MG-ADL responders compared with one (14%) in seven in the placebo group. More specifically, participants who received a placebo reported a decrease in MG-ADL of 2.28 points, while those who received rozanolixizumab reported increases of 7.28 points at a dose of 7 mg/kg and 4.16 points at a dose of 10 mg/kg. As early as day 8, rozanolixizumab caused a rapid decrease in IgG levels that was associated with improvements in efficacy results. Headache, diarrhea, fever, and nausea were the most commonly reported treatment-associated adverse events (187).

All these encouraging results have led to a priority review of the FDA’s Biologic License Application (BLA) and advance efforts to approve rozanolizizumab for the treatment of adults with generalized MG (gMG) who are AChR or MuSK antibody positive. All ongoing studies on the use of FcRn inhibitors in MuSK-MG are summarized in **Table 3**.

### 6.5 Chimeric autoantibody receptor T cells

The novel technology of chimeric auto-antibody receptor T (CAAR-T) cells involves genetically engineered endogenous T cells and subsequent expansion. The patient is then given autologous T cells, which detect antigen-specific B cells that are carrying the BCR against the MuSK antigens and cause pathogenic B cells to undergo apoptosis.

Phase I and II studies employing CD8-positive CAR T immunotherapy against plasma cells that express BCMA are currently being conducted.

Another ongoing clinical study is investigating the different dosing regimens of MuSK-CAART alone, in combination with cyclophosphamide (CY), and in combination with CY and fludarabine (FLU) under number NCT05451212 and is still recruiting patients. Recent findings showed that in an EAMG animal model, MuSK-CAART lowered anti-MuSK IgG without affecting B-cell or total IgG levels, indicating MuSK-specific B-cell depletion (161). MuSK-CAART is considered a cellular precision immunotherapy in the field of autoantibody-mediated neurological autoimmune diseases **(** **Table 3** **).**

## 7 Pregnancy in MuSK Myasthenia Gravis

Evidence for the emergence of MuSK-MG during pregnancy is sparse and most information comes from case reports. Severe MG exacerbations have been reported during pregnancy, especially in newly diagnosed patients and not being stable under treatment (188, 189). In general, short disease duration and severe disease have been recognized as risk factors for MG worsening during pregnancy, whereas previous thymectomy is protective (190, 191). One Portuguese study looked back at the pregnancy history of 17 MuSK-positive women, 13 of whom had more than one pregnancy (27 total pregnancies studied) (192). Upon the time of conception, all were on steroids, with one on azathioprine and another on IVIg maintenance infusions. Only mild pregnancy-related changes were noted, some requiring dosage adjustments. No changes were counted as recurrences.

Pregnancy studies using mycophenolate mofetil, methotrexate, and cyclosphasmamide in animals or humans have revealed fetal malformations. As a result, the possible benefit to pregnancy is exceeded by the danger (193). Regarding azathioprine, the benefit may outweigh the risk and could be used with close monitoring of the fetus. Corticosteroids are recommended to be used at the lowest possible dose during pregnancy (194). Existing data on pregnancy outcomes following RTX can frequently be affected by the use of possibly teratogenic medications and other underlying medical problems (195). According to the prescribing information, women of childbearing potential and non-sterile men should be encouraged to use effective contraception for 12 months after the last dose of RTX.

Plasmapheresis might be used to treat severe disease exacerbations during pregnancy. Plasmapheresis is considered to create fluid changes that might cause hypotension and potentially jeopardize the pregnancy; hence the mother and fetus must be closely monitored. In cases refractory to steroids and when plasmapheresis is unavailable, IVIG can be used during pregnancy; with this approach, MuSK patients have shown a favorable response and, based on current evidence, mothers can continue breastfeeding. A large Italian study examined the consequences of 936 plasmapheresis procedures performed during 57 pregnancies; treatment rationale varied and among others included some for MG etiologies. Just 2% had serious adverse effects, and none required hospitalization or continued hospitalization (196).

## 8 Final considerations

Even though great progress has been made in the field of MuSK immunopathology, unanswered questions still exist. Are IgG4 B cells effective in producing LLPCs? The inadequate response to rituximab in specific cases could suggest that some LLPCs most probably do exist in MuSK-MG patients. Whether the dominance of IgG4 antibodies targeting MuSK is caused by a genetic predisposition to generate ubiquitously IgG4 responses is unknown. We currently do not know when during the clinical course of the disease there is an active MuSK-IgG subclass switch, especially towards IgG4, and whether there is a longitudinal germinal center activity. A MuSK-MG patient was described to undergo a class switch from IgG4 antibodies to IgG1 MuSK antibodies whilst entering stable remission and we currently don’t know what drives this conversion, a knowledge that could open new therapeutic strategies (50). Future studies are needed to assess the role of post-translational modifications such as galactosylation on the pathogenic profile of IgG4 antibodies (activate complement, Fab arm exchange capacity).

The pathogenetic role of antibodies against MuSK has been demonstrated in a passive transfer animal model with immunoglobulins isolated from the serum of MG patients or with recombinant antibodies isolated from B or plasma cells isolated from the periphery of MG patients. *In vitro* studies of IgG pathogenicity have also been performed but animal models provide a more complex picture of the role of these antibodies *in vivo*. Recent studies revealed that the functional monovalency of IgG4 MuSK MG antibodies is crucial for inducing myasthenia (77). In-vitro-produced recombinant antibodies (all subclasses) usually possess bivalent forms (monospecific and have not undergone Fab-arm exchange) and act as partial MuSK agonists, as they induce MuSK kinase activity through phosphorylation, dimerize MuSK, promote AChR clustering and, variable responses in mice causing no or a mild myasthenic phenotype (less pathogenic profile) (80). When compared to the natural agonist agrin, the agonistic bivalent patient-derived MuSK monoclonal antibodies did not induce AChR clustering in the C2C12 myotube assay to the extent as agrin (52, 77). Mice exposed to bivalent 13–3B5 monoclonal antibody exhibited a mild clinical phenotype, with substantial loss of AChR in the NMJs and subclinical myasthenia. The 13–3B5 also induced smaller clusters compared with 11–3F6, another bivalent antibody (80).

One study produced recombinant antibodies that bound the Ig-like 2 of MuSK and promoted MuSK activation and interestingly displayed an inhibitory effect on MuSK signaling (52). This alternative mechanism for inhibiting AChR clustering with recombinant bivalent antibodies needs further studies and animal models could further elucidate the ensuing pathogenetic mechanisms (129). On the other hand, bispecific, functionally monovalent IgG4 anti-MuSK antibodies diminished MuSK signaling and subsequent AchR clustering. These antibodies are mainly found in their native conformation in patient peripheral blood, whereas recombinant produced monovalent IgG4 are produced *in vitro* with either controlled Fab-arm exchange (cFAE) methodology or by papain digestion (production of Fabs—simulating Fab-arm exchanged IgG4), something that makes it difficult to assess the circulating pathogenic autoantibodies in the serum of MG patients (77, 80). *In vitro*, these Fab fragments inhibited agrin-dependent MuSK phosphorylation and AChR clustering similar to patient serum-derived MuSK IgG4 (80).

Patient MuSK IgG1–3 antibodies (not recombinant antibodies) do not affect MuSK-Lrp4 interaction, but reduce agrin-induced AchR clustering in cultured myotubes (51). Collectively, MG exacerbation and clinical severity will rely on combined action of antagonistic and agonistic effects, that are determined to a significant proportion by antibody monovalency. We cannot rule out that complement activation could occur and lead to pathogenicity but this seems less likely and needs further studies with IgG1-3 bivalent monoclonal antibodies.

Interestingly, outside MG pathology, the promotion of MuSK activation constitutes a promising therapeutic strategy for other diseases such as amyotrophic lateral sclerosis (ALS) (208). A recent study developed different agonist antibodies binding the MuSK Ig-like 1 domain that even though *in vitro* experimental settings exhibited a beneficial effect associated with MuSK activation, in mice models this effect was not found (sudden death due to urologic syndrome) (209). Of interest, forced activation of MuSK signaling holds therapeutic promise in neuromuscular disorders characterized by NMJ deficits. So, in MuSK MG some bivalent antibodies that stimulate MuSK signaling may have a protective role towards others with pathogenic profiles and the balance among them may determine the clinical severity and exacerbation.

It seems that antibodies with different effector mechanisms may coexist and agonistic or blocking functions apart from valency and antigen binding avidity could be also controlled by the IgG subclasses, the corresponding Fc-FcγR interactions, and the ability for complement activation. Of note, an IgG4 monoclonal antibody targeting acetylcholine receptor (AChR) diminished subsequent complement-mediated tissue damage induced by IgG1 directed to AChR in a passive transfer model of MG (129). Nevertheless, this is not the case for all diseases with implicated IgG4 antibodies. A recent study showed that while IgG switching to IgG4 subclass is a protective mechanism in IgG4-mediated autoimmune diseases with anti-ADAMTS13 autoantibodies (TTP; thrombotic thrombocytopenic purpura), this is not relevant for Pemphigus foliaceus (PF) in which anti-Dsg1 (desmoglein-1) IgG4 subclass exacerbates the pathogenicity in anti-Dsg1 autoantibodies (210). These differences were attributed to the magnitude of IgG subclass and Fc-FcγR interaction, leading to different functions regarding the clearance of autoantibody-Ag complexes.

Most evidence exists to support the presence of specific memory and short-lived plasma cells (SLPB) as the main producers of MuSK IgG. B- cell or plasma cell infiltrates were not infiltrating the neuromuscular junctions of intercostal muscles in MuSK-MG patients and germinal center-like structures are not found in the thymus of MuSK patients (41, 89, 211, 212). Nevertheless, there are still some patients refractory to anti-CD20 treatment. On the other hand, little evidence exists for drugs targeting LLPCs such as bortezomib, which was found effective in difficult-to-treat MuSK-MG patients, not responding to RTX. Knowledge of anti-CD19 treatment efficacy, which has been approved for NMOSD, is still lacking for MuSK-MG, and ongoing trials are assessing this promising treatment option. Another difficult question not entirely answered is the proper time for re-treating MuSK patients with anti-CD20 therapies for long-term remission and after how many re-doses a patient is considered refractory. Assessing the reemergence of CD19+ or CD27+ cells has been proposed as a peripheral biomarker for helping clinicians to proper disease control minimizing the cumulative dose of RTX. For all above, further studies are needed before reaching definite conclusions.

It seems that, in contrast to autoimmune disorders caused by IgG1 or 3 antibodies, neurological IgG4-antibody-mediated diseases share a particular/common disease mechanism. Rituximab, a monoclonal antibody that targets CD20 with the exception of stem cells, pro-B cells, and plasma cells, has been demonstrated to exhibit favorable effects on the treatment of LGI1 limbic encephalitis, MuSK-MG, CIDP, and pemphigus. Novel treatment strategies are being developed and bring hope, especially for patients refractory to anti-CD20 agents. The current knowledge of MuSK-MG pathophysiology with the expanding role of specific B cells in the pathogenetic process opened the way for more targeted approaches (anti-CD19, CD38). Ongoing clinical trials are currently recruiting patients and assessing the efficacy of BTK inhibitors (tolebrutinib) and humanized mAb targeting both cells- surface-bound and soluble IL6 receptor agents (satralizumab, tocilizumab). Novel agents may in the future lower the need for plasmapheresis in difficult-to-treat patients and subcutaneous injections of FcrN blockers could bring a revolution in daily clinical practice. IgG, for a number of additional neurological conditions, such as autoimmune encephalopathies, NMOSD, and inflammatory neuropathies, FcRn-targeted treatments are now being investigated in clinical studies. One limitation of the blockage of FcRn receptors is the non-specific elimination of pathogenic.

An innovative method that has been tried on animal models of EAMG utilizes an absorber column to selectively remove antigen-specific antibodies and then depleted blood is reinfused (213, 214). Significant portions of the autoantibodies are being removed, resulting in significant symptom relief. In the decade that precision medicine is the optimal goal in treatment options, the specific targeting of pathogenic cells expressing the autoantigen and leaving alive all other B cells could become a revolutionary treatment choice, and we are not far away from this.

## Author contributions

AV and EK: first drafting and editing. AV: image making. JT: concept, design, drafting, and editing. All authors contributed to the article and approved the submitted version.
